# Modulation of Dendritic Cell Immunobiology via Inhibition of 3-Hydroxy-3-Methylglutaryl-CoA (HMG-CoA) Reductase

**DOI:** 10.1371/journal.pone.0100871

**Published:** 2014-07-11

**Authors:** Tina Leuenberger, Caspar F. Pfueller, Felix Luessi, Ivo Bendix, Magdalena Paterka, Timour Prozorovski, Denise Treue, Sarah Luenstedt, Josephine Herz, Volker Siffrin, Carmen Infante-Duarte, Frauke Zipp, Sonia Waiczies

**Affiliations:** 1 Department of Neurology, Focus Program Translational Neuroscience (FTN), Rhine Main Neuroscience Network (rmn^2^), University Medical Center of the Johannes Gutenberg-University of Mainz, Mainz, Germany; 2 Max Delbrueck Center for Molecular Medicine Berlin-Buch, Berlin, Germany; 3 NeuroCure Clinical Research Center, Charité University Medicine Berlin, Berlin, Germany; 4 Department of Pediatrics I/Neonatology, University Hospital Essen, Essen, Germany; 5 Department of Neurology, Heinrich-Heine-University, Duesseldorf, Germany; 6 Institute of Pathology, Charité University Medicine Berlin, Berlin, Germany; 7 Institute for Medical Immunology, Charité University Medicine Berlin, Berlin, Germany; 8 Berlin Ultrahigh Field Facility (B.U.F.F.), Max Delbrück Center for Molecular Medicine, Berlin, Germany; University of Münster, Germany

## Abstract

The maturation status of dendritic cells determines whether interacting T cells are activated or if they become tolerant. Previously we could induce T cell tolerance by applying a 3-hydroxy-3-methylglutaryl-CoA (HMG-CoA) reductase inhibitor (HMGCRI) atorvastatin, which also modulates MHC class II expression and has therapeutic potential in autoimmune disease. Here, we aimed at elucidating the impact of this therapeutic strategy on T cell differentiation as a consequence of alterations in dendritic cell function. We investigated the effect of HMGCRI during differentiation of peripheral human monocytes and murine bone marrow precursors to immature DC *in vitro* and assessed their phenotype. To examine the stimulatory and tolerogenic capacity of these modulated immature dendritic cells, we measured proliferation and suppressive function of CD4+ T cells after stimulation with the modulated immature dendritic cells. We found that an HMGCRI, atorvastatin, prevents dendrite formation during the generation of immature dendritic cells. The modulated immature dendritic cells had a diminished capacity to take up and present antigen as well as to induce an immune response. Of note, the consequence was an increased capacity to differentiate naïve T cells towards a suppressor phenotype that is less sensitive to proinflammatory stimuli and can effectively inhibit the proliferation of T effector cells *in vitro*. Thus, manipulation of antigen-presenting cells by HMGCRI contributes to an attenuated immune response as shown by promotion of T cells with suppressive capacities.

## Introduction

Bidirectional interactions between dendritic cells (DC) as professional antigen-presenting cells (APC) and T cells may result in either promotion or suppression of immune responses, depending on the environmental cues. In the peripheral circulation resting or immature DC (iDC) have a high capacity for taking up antigen but low capacity for binding and stimulating T cells [1]. In the presence of an inflammatory milieu, iDC transform into mature DC that exhibit a limited capacity for taking up antigen but exceptional capacity at stimulating T cells [2], [3]. In the absence of maturation stimuli, DC remain inactivated at a steady state in peripheral tissues and within lymphoid tissues are able to present MHC-peptide complexes, also at a steady state, to naive T cells. The repetitive division of these T cells ultimately results in their demise since they undergo deletion, giving rise to a state of tolerance [4]. Monocyte-derived iDC were shown to induce a population of anergic T cells with suppressive functions *in vitro* [5] and *in vivo* in both mice [6], [7] and healthy human individuals [8]. Indeed, one strategy introduced at the turn of this century was to expose autologous DC to antigen in the absence of a maturation signal and then transplant them back to induce regulatory T cells *in vivo* [8], [9]. However, one evident problem in applying iDC as clinical therapy in allergy, autoimmunity or transplantation is that the inflammatory environment might lead to DC maturation, which would promote an immune reactivation rather than the desired down-modulation of the immune response. Thus, one clinical approach is to engineer tolerogenic DC *ex vivo* via pharmacological manipulation of these cells during or after their generation to iDC with stable tolerogenic properties. One group of drugs that inhibits the maturation status of differentiated iDC are the 3-hydroxy-3-methylglutaryl-CoA (HMG-CoA)-reductase inhibitors (HMGCRI), a family of cholesterol-lowering drugs also known as statins [10], [11]. We as well as other researchers have previously shown that the HMGCRI atorvastatin is therapeutic in EAE, the animal model of MS [12], [13]. The first EAE study reported on a reduction in Th1 differentiation in myelin-reactive CD4+ T cells following atorvastatin treatment as well as a regulation of the APC compartment, which subsequently influenced the T cell response [13]. Our group reported on a direct influence of atorvastatin on the anergic status [14] and cytoskeletal reorganization [15] of T cells as possible mechanisms for the salutary role of atorvastatin treatment.

Here we focused on the capacity of atorvastatin in modulating the initiation of the immune response by exploring the influence of this drug during the generation of iDC from peripheral human monocytes or murine bone marrow precursors. We report that atorvastatin can – via morphological alterations – interfere with early differentiation processes, resulting in iDC (which we refer to as aiDC) less capable of antigen uptake and as a result less competent in stimulating allogeneic T cells. These observations were accompanied by an inhibited surface expression of costimulatory molecules and maturation markers. Furthermore, aiDC showed a pronounced ability to transform naïve CD4+ T cells into suppressor cells that have an increased inhibitory capacity on activated T cells. Our present data demonstrate the potential of atorvastatin to induce an anergic regulatory T cell phenotype via alterations within the professional antigen-presenting cell compartment.

## Materials and Methods

### Reagents and antibodies

Pure atorvastatin (provided by Pfizer) was dissolved in PBS. Mevalonate – metabolite product of HMG-CoA reduction – was prepared, as already described, by activating L-mevalonic acid lactone (Sigma) [12]. Con A was purchased from Sigma. OVA_323–339_ peptide was synthesized by P. Henklein's group, Department of Biochemistry, Charité University Medicine Berlin. Recombinant human IL-2 (Hoffmann-La Roche), recombinant human IL-10 (Sigma), Interferon-α-2a (Roferon^®^,Roche), recombinant human IL-15 (PeproTech EC), recombinant human GM-CSF (R&D). Anti-CD3/OKT3 (kindly provided by Janssen-Cilag), anti-CD28 (R&D Systems).

### Peripheral immune cells

PBMC were isolated by Ficoll Hypaque density gradient centrifugation from buffy coats (German Red Cross, Berlin) of healthy donors taken in accordance with the local ethics committee (Ethikkommission der Charité, Universitätsmedizin Berlin, Germany). The ethics committee waived the need for consent due to fact that buffy coats of anonymous healthy blood donors were provided by the German Red Cross donation center, Berlin, Germany.

### Mice

C57Bl/6 and SJL/N mice were obtained from Charles River Laboratories. Beta-actin EGFP-C57BL/6 (C57BL/6-Tg(ACTB-EGFP)1Osb/J), beta-actin RFP-C57BL/6 (C57BL/6 *Rosa26 tdRFP* “*ΔNeo-flip*”, obtained from H.J. Fehling, Ulm) and OT-II (C57BL/6-Tg(TcraTcrb)425Cbn) mice were bred under specifically pathogen free (SPF) conditions at the central animal facility of the Charité – Universitaetsmedizin Berlin (FEM). All animal experiments were approved by the appropriate state committees for animal welfare (Landesamt für Gesundheit und Soziales (LAGeSo), Berlin, Germany).

### ^3^H-thymidine incorporation assay

To measure the level of T cell response, cultures were cultured for three days in 96-well round-bottom plates, followed by incubation for 18 h with [^3^H] thymidine (Amersham) at a final concentration of 100 µCi/ml. [^3^H] thymidine incorporation was measured in a β-scintillation counter (Microbeta; Wallac). Results (means of triplicate cultures) were expressed as counts per minute (cpm) and T cell response calculated as an index of stimulation following alloantigen or unspecific stimulus: cpm_stimulated_/cpm_unstimulated_.

### Generation of human dendritic cells

Monocytes were sorted from PBMC from healthy donors using human CD14 microbeads (Miltenyi Biotec). The purity of the monocyte fraction was checked by flow cytometry using an APC-labeled anti-human CD14 antibody (BD Pharmingen). The resulting CD14 positive monocyte fraction was cultured at 4 * 10^6^ cells/ml in the presence of recombinant human GM-CSF (50 ng/ml) and recombinant human IL-4 (20 ng/ml) and different concentrations of atorvastatin. On day 3 fresh medium, GM-CSF, IL-4 were supplemented. On day 7 generated dendritic cells were harvested.

### Generation of mouse dendritic cells

Femurs of C57Bl/6 or beta-actin-EGFP-B6 mice were aseptically removed. BM cells were isolated by flushing femurs with PBS containing 0.5% BSA, the cells were grown in 100 mm Petri dishes in a RPMI-1640 with and different concentrations of atorvastatin, penicillin, streptomycin, glutamine, 2-mercaptoethanol and 10% heat-inactivated FBS (Biochrom Germany) supplemented with GM-CSF containing supernatant from a transfected 293FT HEK cell line. GM-CSF concentration of the supernatant was measured by ELISA, normalized and used in a final concentration of 10 ng/ml. On days 3, 6 and 8 fresh medium and GM-CSF supernatant were added; dendritic cells were harvested on day 10.

### Measurement of polymerized actin (f-actin) by immunofluorescence staining and flow cytometry

For microscopic analysis, immature dendritic cells were plated on poly-L-ornithine (Sigma) coated glass cover slips for 2 h at 37°C. Cells were then rinsed twice with PBS, fixed with 4% PFA and stained with rhodamine-coupled phalloidin (1:50 dilution, Molecular Probes). Nuclei were stained with 1 µg/ml Hoechst 33342 (Sigma). Preparations were visualized by using an inverse fluorescence microscope (Leica, Heidelberg, Germany) for triple immunofluorescence. Human iDC were also stained with a biotin-coupled anti-CD11c as primary and FITC as secondary antibody (BD Pharmingen). Five random fields from each section were viewed under a 20× objective and a representative field was depicted. Thresholds were set to eliminate background fluorescence if present. For further quantitative analysis we applied a flow-cytometric protocol as previously described [15]. Briefly, murine immature dendritic cells were washed with PBS and thereafter fixed with 2% PFA for 15 min at room temperature in the dark. After thoroughly washing with PBS, cells were permeabilized with saponin buffer (0.5% saponin, 0.5% BSA) for 10 min and then resuspended in staining solution containing 50 ng/ml FITC-Phalloidin (Sigma). After 45 min incubation at room temperature in the dark, cells were thoroughly washed again and mean fluorescence intensities were measured using a FACSCalibur flow cytometer (BD Biosciences) and analyzed with CELLQuest software.

### Flow cytometry

Cell surface molecules were analysed by a FACSCalibur^®^ flow cytometer (Becton Dickinson). Cells were incubated for 10 min at 4°C with relevant or control antibodies in 100 µl total staining volume in PBS-BSA. After staining, the cells were washed and resuspended in 300 µl of PBS-BSA. The following antibodies were used for the staining of human monocytes, T cells and dendritic cells:, anti-CD1c-FITC [*anti-BDCA-1*] (Miltenyi), anti-CD11b-PE [*Mac-1*] (BD Pharmingen), anti-CD14-APC (BD Pharmingen), anti-CD40-FITC (BD Pharmingen), anti-CD45RA-APC (BD Pharmingen), anti-CD86-PE (eBioscience), anti-HLA-DR-CyChrome (BD Pharmingen). The following antibodies were used for the staining of murine dendritic cells and T cells: CD4-AF647, vα2-Bio, SA-PacificBlue, CD11c-FITC, CD86-PE, MHC II was measured by a combination of biotin-labeled I-A^b^/SA-APC (all BD Pharmingen). To measure proliferation, T cells were labeled with the fluorescent dye CFSE (Molecular Probes).

### Phagocytosis measured by FITC-dextran incorporation

To measure the phagocytotic activity of iDCs, human iDCs (1×10^5^) were resuspended in 100 µl PBS containing 1% human AB serum and incubated with FITC-dextran (Sigma, 1 mg/ml) at 37°C and 0°C (negative control) for 30 min. The incubations were stopped by adding 2 ml ice-cold PBS containing 1% human serum and 0.02% sodium azide. The cells were washed three times with cold PBS-azide and analyzed on a FACSCalibur flow cytometer. Appropriate gates were set for the analysis of viable cells to exclude debris and dead cells.

### Mixed leukocyte reaction

In the human system immature dendritic cells were co-cultured with varying ratios of allogeneic CD4+ T cells (iDC alone, 1:10, 1:20, 1:40, 1:80), isolated from healthy donors using the CD4 T cell isolation kit (Miltenyi Biotec). In the mouse system C57Bl/6 derived iDC were co-cultured with varying ratios of spleen cells from SJL/N mice (iDC alone, 1:10, 1:20, 1:40). Strength of MLR was measured by [^3^H]-thymidine incorporation in both systems.

### Generation of human regulatory T cells

Naïve CD4 T cells were sorted from PBMC from healthy donors using a naïve CD4+ T cell isolation kit (Miltenyi) and were co-cultured with previously generated allogeneic iDC at a ratio of 20:1 at a resulting cell concentration of 1 million cells per ml in RPMI-1640 containing 50 IU/ml penicillin, 50 µg/ml streptomycin, 2 mM glutamine and 10% FBS (Biochrom). On day 0 a cytokine cocktail (concentrations given in parentheses) consisting of recombinant IL-10 (100 U/ml), interferon-α-2a (675 U/ml), IL-15 (20 U/ml) and IL-2 (16 U/ml) was added to the culture. Every three days fresh medium, IL-2 and IL-15 were supplemented. On day 7 cells were restimulated with allogeneic dendritic cells and cytokines as on day 0 and cultured for additional 7 days. This protocol is adapted from Jonuleit et al. [5] and Bacchetta et al. [16].

### *Ex vivo* restimulation of lymph node T cells

Immature dendritic cells derived from bone marrow of beta-actin RFP-transgenic C57BL/6 mice generated in the absence or presence of atorvastatin were incubated for 3 h with OVA_323-339_ peptide (100 µg/ml) and then injected intracutaneously into Rag1-ko mice. Naïve CD4 T cells were isolated from the spleen and lymph node cells of OT-II animals by magnetic cell sorting, using the Naïve T cell isolation kit (Miltenyi Biotec), and transferred to the Rag1-ko mice one day after the iDC. After 5 days cells were isolated from the draining lymph nodes, restimulated *in vitro* with varying concentrations of OVA-peptide or control stimuli (anti-CD3/CD28, concavalin A), and proliferation was measured in a standard ^3^H-thymidine incorporation assay.

### Priming of antigen-specific T cells

Immature dendritic cells (iDC and aiDC) were incubated with OVA_323–339_-peptide for 30 min at 37°C. Cells were then harvested, washed thoroughly, and cocultured with CFSE-labeled OVA-specific naïve T cells isolated from OT-II transgenic mice by magnetic cell sorting, using the Naïve T cell isolation kit (Miltenyi Biotec). The ratio of iDC/aiDC to T cells was 1:10. After 72 hours cells were harvested, T cell markers were stained (CD4-AF647, vα2-Bio, streptavidin-PacificBlue) and T cell proliferation was measured by FACS as a decrease in CFSE-intensity.

### Intracellular staining for IL-10 expression

After the differentiation period of 10 days murine iDC were harvested, left unstimulated or stimulated with PMA and ionomycin for 6 hours. After 4 hours brefeldin A was added to the culture. The cells were then washed, surface stained with CD11c, fixed with 2% PFA, and stained for intracellular IL-10 expression (anti-IL-10-APC, BD Pharmingen) and analysed by flow cytometry on a FACSCanto II flow cytometer.

### Measurement of cytokines from supernatants of DC-cultures

To measure secretion of cytokines by DC generated in the absence or presence of atorvastatin, murine and human iDC/aiDC were generated as described above. Immature DC were harvested and replated in a defined concentration with LPS, but no cytokines. After 24 hours, supernatants were collected and used to determine cytokine concentrations using the mouse/human FlowCytomix Multiplex kit from eBioscience, according to manufacturers instructions.

### Suppression assay

Following 14 days differentiation with allogeneic iDC, human regulatory T cells were harvested and co-cultured with anti-CD3/anti-CD28 preactivated autologous CD4 T cells that were prepared using CD4 microbeads (Miltenyi Biotec) in varying ratios. Total cell numbers per well were kept constant at 2×10^5^. Inhibition of proliferation of preactivated CD4 T cells was detected by a standard ^3^H-thymidine incorporation assay.

### In vivo migration assay

Immature dendritic cells (iDC and aiDC) cells derived from bone marrow of beta-actin RFP-transgenic C57BL/6 mice were incubated with LPS for 12 h and OVA_323-339_ peptide for 3 h. Thereafter, RFP-DC were harvested, washed thoroughly in serum-free buffer and administered intracutaneously (8×10^6^) into the hind limb of C57BL/6 mice. Following 18 h, mice were sacrificed; lymph nodes extracted and transplanted RFP+CD11c+CD11b+ cells measured by FACS.

### Data analysis

**Figure 1:**
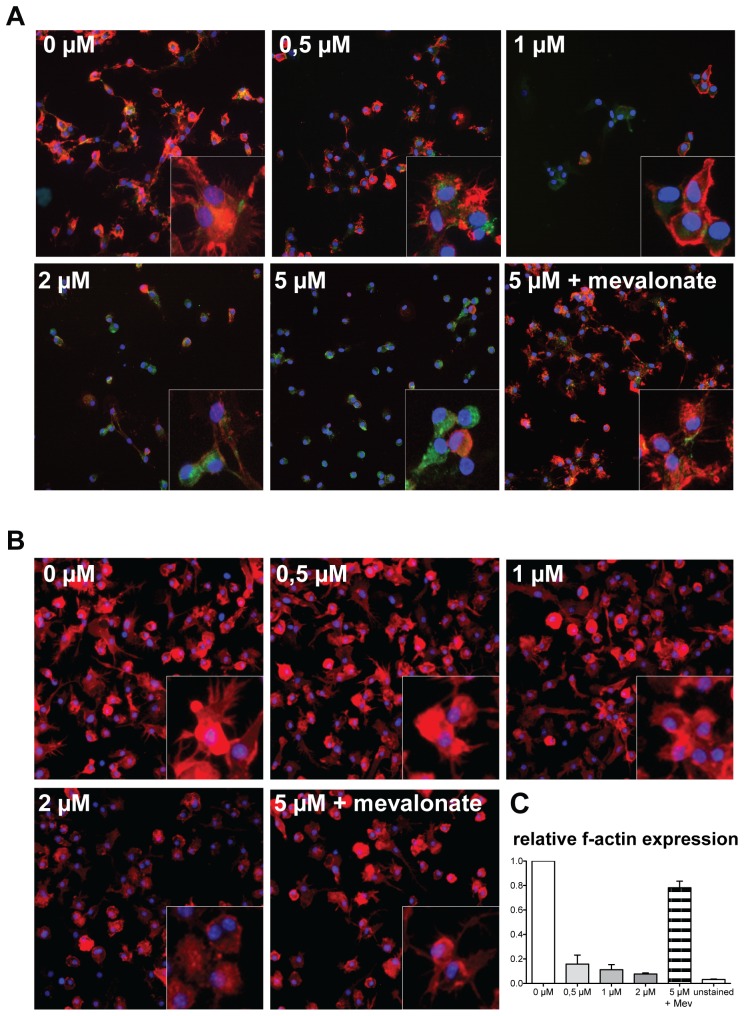
Influence of atorvastatin treatment during DC differentiation on f-actin expression. Human monocytes and murine BMC were differentiated into iDC in the presence of different atorvastatin concentrations: human iDC (A) untreated, 0.5 µM, 1 µM, 2 µM, 5 µM and 5 µM atorvastatin in the presence of 200 µM mevalonate. Murine iDC (B) untreated, 0.5 µM, 1 µM, 2 µM and 5 µM atorvastatin in the presence of 200 µM mevalonate. DC were stained for f-actin with rhodamin-coupled phalloidin (red) and nuclei were stained with Hoechst 33342 (blue). Human iDC were additionally stained for CD11c (green). The insert panels highlight representative structural cell features at an additional threefold magnification. (C) The level of actin polymerization in murine iDC was quantified by flow cytometry. The fluorescence intensity of phalloidin-FITC bound to f-actin was analyzed for different atorvastatin concentrations and mevalonate. The data show relative f-actin expression compared to untreated iDC derived from five independent experiments (mean + SD).

**Figure 2:**
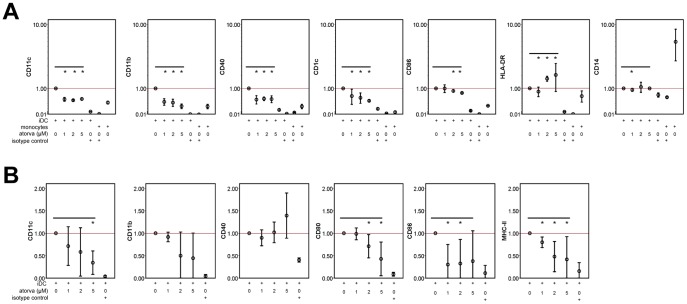
Expression of MHCII, costimulatory and maturation markers is attenuated in aiDCs. (A) Expression of surface markers CD1c, CD11b, CD11c, CD14, CD40, CD80 and CD86 on human iDC cultured in the presence of atorvastatin was measured by flow cytometry after the differentiation period (day 7) and compared with surface marker expression of untreated iDC and undifferentiated monocytes from peripheral blood together with isotype controls for untreated iDC and monocytes. The data show relative expression of surface molecules compared to untreated iDC derived from three independent experiments (mean ± SD). (B) Expression of surface markers CD11b, CD11c, CD40, CD80, CD86 and MHC class II of murine iDC cultured in the presence of atorvastatin was measured by flow cytometry after the differentiation period (day 10) and compared with surface marker expression of untreated iDC and isotype controls for untreated iDC. The data show relative expression of surface molecules compared to untreated iDC derived from three independent experiments (mean ± SD).

**Figure 3:**
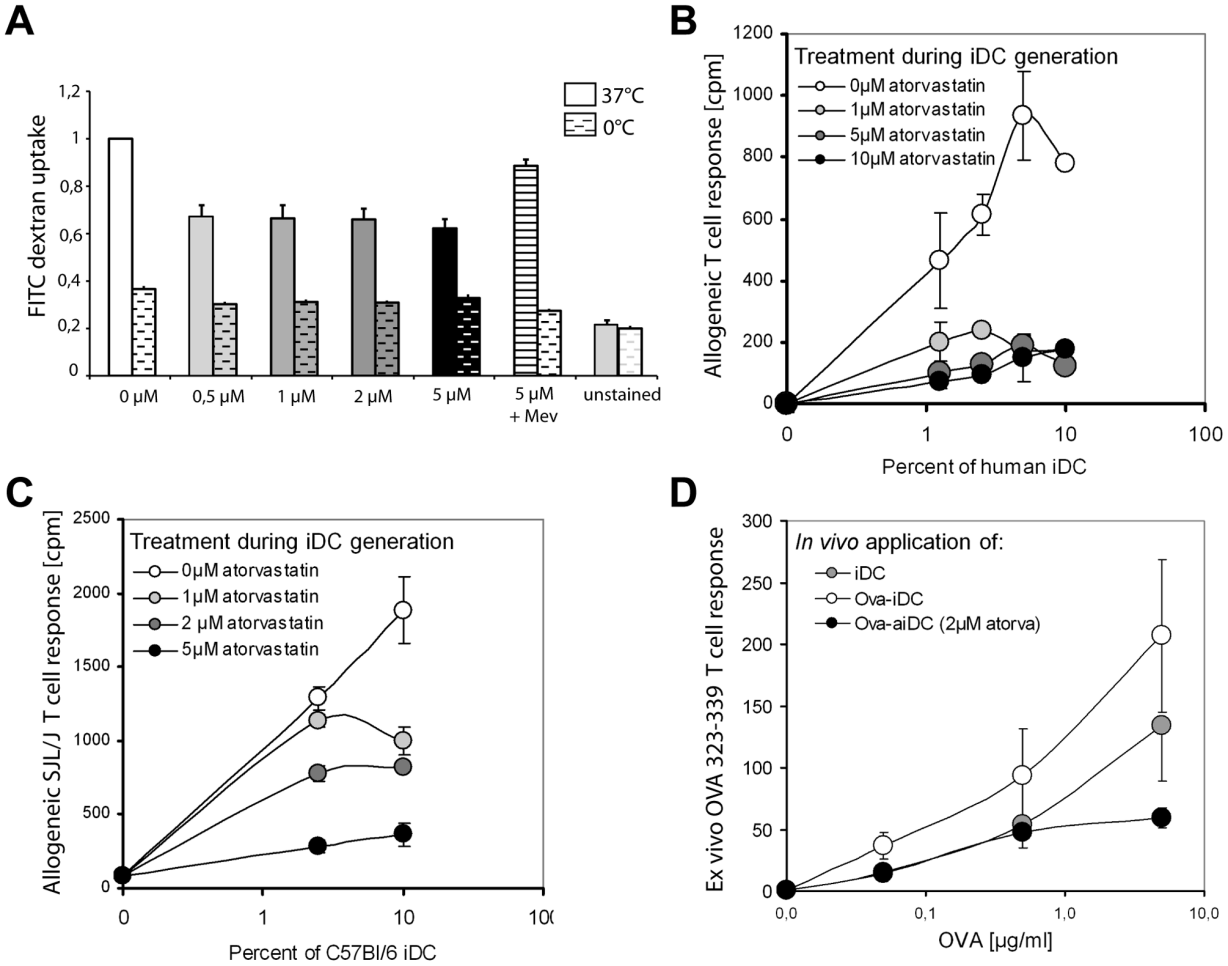
iDC generated in the presence of atorvastatin have a decreased capacity to take up antigen and to initiate an allogenic response. (A) FITC-dextran uptake was measured in human iDC by flow cytometry. Data show the mean relative fluorescence values for different atorvastatin concentrations compared to untreated iDC (mean + SD) for FITC-dextran uptake at 37°C (left columns) and 0°C (right dashed columns). (B) Human iDC were generated from monocytes in the presence of different atorvastatin concentrations and subjected to MLR with allogeneic T cells in different iDC-to-T cell ratios (iDC alone, 1:10, 1:20, 1:40, 1:80). MLR strength was measured by ^3^H-thymidine incorporation of the co-culture. Plotted is the mean ^3^H-thymidine incorporation in counts per minute (cpm), these data are representative of three independent experiments. Data are representative of three independent experiments. (C) Mouse iDC (C57Bl/6) were generated from BMC in the presence of different atorvastatin concentrations and subjected to MLR with allogeneic spleen cells (SJL/N) in different iDC to spleen cell ratios (iDC alone, 1:10, 1:20, 1:40). MLR strength was measured by ^3^H-thymidine incorporation of the co-culture. Plotted is the mean ^3^H-thymidine incorporation in counts per minute (cpm), these data are representative of three independent experiments. (D) Ova-loaded iDC and aiDC were injected subcutaneously. Ova-specific OT-II T cell response of draining lymph node cells was measured by ^3^H-thymidine incorporation. Plotted are the mean stimulation indices as ratio of ^3^H-thymidine incorporation compared to unstimulated lymph node cells alone. Data are representative of three independent experiments.

**Figure 4:**
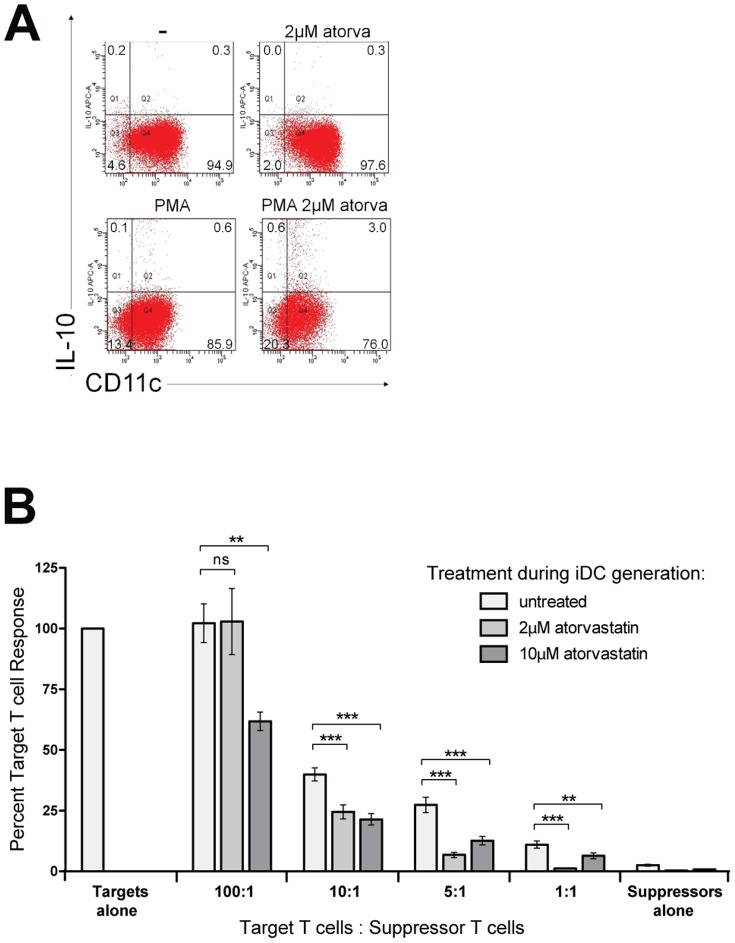
Atorvastatin primes iDC to produce IL-10 and to generate T cells with a greater suppressive capacity. (A) Intracellular IL-10 expression in C57Bl/6-derived iDC and aiDC, unstimulated and stimulated with PMA-ionomycin, was measured by flow cytometry. Quadrants were set according to unstained controls. (B) Atorvastatin treated and untreated iDC were used to generate a regulatory T cell population by repetitive stimulation of allogeneic naïve CD4 T cells in the presence of regulatory cytokines. The regulatory capacity was assessed by the suppression of proliferation of pre-activated CD4 effector cells. Shown are the changes of effector T cell proliferation (in percent) for different suppressor T cell to T effector cell (targets) ratios and different atorvastatin concentrations (untreated, 2 µM and 10 µM) used during generation of iDC. Representative experiments of at least three are shown. p<0.05 was considered statistically significant, asterisks represent ** = p<0.01, *** = p<0.001.

Relative f-actin expression compared to untreated iDC in Fig 1 is presented as MEAN +/− SD. Relative expression of surface molecules in Fig 2 is presented as MEAN +/− SD. To calculate statistical significances between untreated iDC and aiDC, we used the Mann-Whitney-U-test with respect to the numbers of experiments performed; p<0.05 (*) was considered statistically significant. Proliferation data in Fig 3 and 4 are presented as MEAN +/− SEM. Statistical analysis as shown in Fig 4 was performed with the Kruskal-Wallis-Test and Dunnett's Multiple Comparison Post Test, p<0.05 was considered statistically significant.

## Results

### Cytoskeletal alterations in iDC generated in the presence of atorvastatin

One major hallmark during the generation of dendritic cells is the formation of membrane protrusions, or dendrites, that give the typical terminology to these cells. This shaping involves cytoskeletal changes that require signaling via Rho GTPases [17]. Since these intracellular molecules are targeted by HMGCRI [15], [18], we applied atorvastatin during the differentiation process of myeloid precursor cells to iDC. Indeed even low doses of atorvastatin led to microscopically-visible morphologic changes in as early as 2–3 days of culture. To assess the integrity of the cytoskeleton we investigated the level of polymerized actin (f-actin). For this we stained human (Fig 1A) and murine (Fig 1B) iDC with rhodamine-coupled phalloidin that binds to f-actin. While untreated precursor cells differentiated into cells with membrane protrusions or dendrites, generation in the presence of atorvastatin led to a dose-dependent loss of dendritic spikes; these cells also expressed less f-actin, presented with a more rounded cellular morphology and were less adherent (data not shown). These morphologic changes were not visible when the product of HMG-CoA reduction, mevalonate (200 µM), was present in the differentiation culture. In the human system (Fig 1A) we co-stained for CD11c and found that increasing atorvastatin concentrations also affected CD11c localization; while CD11c is normally diffusely co-expressed with f-actin on cytoplasmic processes and at sites of intercellular contact in DC [19], a more compact and nucleus-associated CD11c signal was observed in aiDC with increasing atorvastatin concentrations (Fig 1A). The flow cytometric analysis of the f-actin expression (Fig 1C) showed a clear atorvastatin concentration-dependent decrease of f-actin and a total reversion of the effect by mevalonate. While lower atorvastatin concentrations up to 2 µM did not alter cell yield significantly, there was a considerable drop at higher doses, especially in murine iDC, also leading to reduced staining quality. We therefore excluded cytochemistry of 5 µM pretreated murine iDC from the analysis. However, this phenomenon could be completely reversed by administration of mevalonate.

Considering the above observations of an altered cytoskeleton in aiDC, we performed *in vivo* migration experiments to determine the migratory capacity of these cells to reach the draining lymph nodes. We show that these cells do not reach the draining lymph node as efficiently as control DC (Fig S1A).

### Changes in surface marker expression in human and murine aiDC

In the earlier atorvastatin EAE study, it was reported that the HMGCRI downregulates the surface expression of typical APC markers, namely MHC class II, CD40 and the costimulatory molecules CD80 and CD86 [13]. Since rearrangements in the actin cytoskeleton are important for the export of surface proteins including MHC-II molecules and costimulatory molecules to the cell surface, we next investigated the expression of markers that are known to be differentially expressed depending on DC maturation state and to be associated with T cell stimulatory capacity. We measured the expression of DC-specific surface markers and costimulatory molecules by flow cytometry. The presence of atorvastatin during the generation of iDC (aiDC) led to a dose-dependent reduction in the surface expression of the typical DC markers CD11b, CD1c (human), CD11c (mouse), and of costimulatory molecules (Fig 2), although in the human system (Fig 2A) differences in CD86 expression were not as pronounced as in the murine system (C57BL/6 mice bone-marrow derived iDC). In the latter we also observed a dose-dependent interference with CD80 and MHC-II surface expression upregulation (Fig 2B). As expected, expression of CD14 was high on monocytes.

Importantly, a complete CD14 down-regulation occurred during the differentiation of monocytes to iDC, irrelevant of atorvastatin treatment. Notwithstanding we still observed a marked decrease in the expression of CD1c and CD11b after atorvastatin incubation, even though these markers are typically up-regulated in parallel to CD14 loss after the differentiation of monocytes to iDC. Undifferentiated monocytes that were used as a negative control, on the other hand, showed only a low expression of CD40, CD86, CD1c and CD11b in comparison to all DC groups (Fig 2A).

### Decreased antigen uptake and presentation by aiDC *in vitro* and *in vivo*

To investigate whether alterations in the iDC cytoskeleton contribute to changes in the capacity of these cells to take up antigen, we incubated iDC with FITC-dextran at 37°C and calculated uptake by measuring fluorescence by flow-cytometric analysis. We observed that increasing doses of atorvastatin led to a decrease in FITC-dextran incorporation in human (Fig 3A) and mouse cells (data not shown), indicating a decrease in antigen uptake by these cells. Next we studied the impact of atorvastatin-mediated interference with antigen uptake and expression of surface molecules on the capacity of iDC to present antigen using a MLR. We first generated iDC from monocytes in the presence or absence of increasing doses of atorvastatin and then co-cultured viable iDC in varying ratios (up to 1:10) with sorted CD4+ cells from a second healthy donor. After 72 h allogeneic culture, the strength of MLR was measured with a ^3^H-thymidine proliferation assay. Maximal allogeneic T cell stimulation was achieved with iDC to T cell ratios of 1:20 (Fig 3B). Priming of iDC with atorvastatin during differentiation from monocytes resulted in a marked dose-dependent decrease in T cell response, with a concentration of 1 µM atorvastatin already leading to significant MLR inhibition. A similar effect was also observed in the murine system (Fig 3C). In this case, we co-cultured bone marrow-derived iDC from C57Bl/6 mice, generated in the presence or absence of atorvastatin with spleen cells of SJL/J mice.

Finally, we assessed the stimulatory capacity of aiDC to prime antigen-specific T cells *in vivo* by measuring T cell proliferation upon *ex vivo* antigen restimulation. We injected iDC and aiDC loaded with OVA_323-339_ peptide intracutaneously into Rag1-ko mice that lack endogenous T cells. The following day we injected mice with naïve CD4 OT-II cells that express a T cell receptor that recognizes the OVA_323-339_ epitope. After 5 days immune cells were isolated from draining lymph nodes, restimulated *in vitro* with OVA-peptide or control stimuli, and proliferation was measured by ^3^[H] incorporation. We show that aiDC fail to promote the typical pronounced T cell response upon antigen-specific restimulation (Fig 3D). These results (Fig 3D) are in line with the *in vitro* MLR experiments with allogenic DC generated in the presence of atorvastatin (Fig 3B, 3C). However, when we performed *in vitro* experiments to study the capacity of aiDC to present OVA-peptide to antigen-specific T cells in culture, we show that aiDC do not significantly differ from control iDC in presenting OVA_323-339_ peptide to naïve CD4 OT-II T cells in vitro (Fig S1B).

### Suppressor activity of *in vitro* generated regulatory T cells is enhanced by aiDC

We have previously reported that the induction of T cell anergy by atorvastatin requires IL-10 signaling [14]. However it has not been clear which immune cell population is the source of this regulatory cytokine. Since IL-10 production by DC has been shown to be critical for the induction of tolerance [20], we next investigated IL-10 levels in iDC generated in the presence of atorvastatin. Indeed aiDC produced significantly higher amounts of IL-10 (up to 9-fold) as assessed by intracellular staining (Fig 4A). We also observed an increased secretion of IL-4 in these cells as well as a decrease in the pro-inflammatory cytokines TNF-alpha, IL-6 and IL-1beta (Fig S2). This is in line with a previous report from a group that studied the influence of simvastatin, another HMGCRI, on already differentiated DC [11].

To investigate the functional relevance of IL-10 producing aiDC in promoting suppressor-like T cells, we generated regulatory T cells via repetitive stimulation of naïve CD4+ T cells with allogeneic iDC. The regulatory capacity of the generated T cells was then evaluated by measuring the extent of the suppressive capacity of these cells on autologous CD4+ effector T cells. We observed that expansion of naïve CD4+ cells in the presence of aiDC resulted in T cells with increased suppressive capacity (Fig 4B). The suppressive effect was strongest for T cells expanded by iDC generated in the presence of 10 µM atorvastatin. However lower doses (2 µM) of atorvastatin still resulted in significant suppression especially at ratios of suppressor to target T cells higher than 1:10.

## Discussion

There has indeed been a longstanding view – stemming particularly from *in vivo* DC-reconstitution assays [21] – that considerable plasticity exists in the development of DC populations. This remarkable degree of plasticity makes these cells ideal therapeutic targets for immune modulation [22], [23]. Indeed the ultimate aim is to transplant or promote the generation of tolerogenic DC in various immune-mediated pathologies [8]. It has previously been reported that the HMGCRI atorvastatin – via a depletion in isoprenoids – inhibits Ras GTPase pathways that are necessary for Th1 differentiation [18] and Rho GTPase pathways that are necessary for proliferation [18] and actomyosin reorganization [15] in myelin-reactive T cells. Here we show that during the differentiation of precursor cells to iDC, atorvastatin precipitates cell structural alterations, similar to those previously reported in T cells [15]. Indeed HMGCRI have already been shown to exhibit an influence on the APC compartment [10], [11], [13], [24].

As the actin-based cytoskeleton is rearranged, it can be assumed that related processes, such as exosome formation and their transport to the cell surface which are required for the presentation of MHC and costimulatory molecules, are altered and result in a diminished surface expression of these molecules independent of their intracellular synthesis. Recently it was shown that different iDC culturing conditions can lead to an altered exosome composition of MHCII and costimulatory molecules [25]. Furthermore exosomes were shown to have immunomodulatory effects in specific contexts [26], [27] and might therefore directly contribute to altered immune responses that add to their role of modulating the surface expression of costimulatory molecules. Notably, we observed that the cellular distribution pattern of CD11c – that is commonly distributed around the f-actin core of podosomes [19] – was more compact and intracellularly aggregated in aiDC. A failure of this integrin to localize to dendritic processes might hinder an optimal and stable contact with interacting T cells. In fact, we could demonstrate that atorvastatin enables iDC to induce a suppressive T cell phenotype. By applying atorvastatin-treated iDC (aiDC) loaded with OVA_323–339_ peptide and OVA-specific T cells in Rag1−/− mice, the capacity to induce less responsive T cells was conserved *in vivo*. The *in vitro*–generated aiDC that induced less responsive T cells expressed less CD40. Ligation of CD40 by CD40L (CD154) on T cells is required for DC to undergo maturation since this signals the induction of the costimulatory molecules CD80 and CD86 that signal back to T cells to boost the immune response [28]. Furthermore, CD40 ligation releases iDC from a “default” control mode by regulatory T cells [29]. On the other hand, iDC incapable of delivering a complete activation signal can induce T cell tolerance via the de novo generation of regulatory T cells [30] or antigen-specific anergy following downregulation of CD154 [31]. Since the CD40/CD154 axis participates in the genesis of inflammatory conditions including atherosclerosis and neuroinflammation [32], [33], manipulation of this system by immunomodulatory agents such as atorvastatin would provide a useful means to shift the T cell response towards a regulatory phenotype. Using a setup adapted from Jonuleit et al. [5] and Bacchetta et al. [16], we showed that the impact of atorvastatin on iDC development renders these cells (aiDC) even more potent at generating anergic suppressor T cells. This setup may not match the cytokine exposure *in vivo*, however we used it here merely as a tool for maintaining and preserving the anergic T cell population. Notably aiDC produced higher amounts of intracellular IL-10. It has previously been reported that iDC can generate anergic IL-10–producing suppressor T cells by repetitively stimulating allogeneic naïve T cells [5]. Our data show that the regulatory T cells generated via repetitive restimulation with IL-10 producing aiDC have more pronounced suppressive properties compared to untreated iDC. Our observation that iDC alone also cause a tolerogenic modulation of effector T cells has already been described in the literature [34]. One proposed mechanism for the suppressor properties of regulatory T cells is the competition for growth factors such as IL-2 between suppressor and effector target cells [35]. From these experiments we cannot exclude competition as one mechanism via which the suppressor cells suppress the target cells. However we kept the total (target + suppressor) cell number constant between the different groups to keep the available growth factors per cell constant. Furthermore, neither iDC nor suppressor T cells alone show any proliferation, thus the competing effects also seem to be negligible.

Our present findings could contribute to the observations made on a reduced severity in T cell mediated-diseases, such as EAE [12], [13], following atorvastatin treatment. In the last ten years there have been several clinical studies in MS patients to investigate the outcome of using HMGRI as stand-alone therapy [36], [37] or as add-on to other disease-modifying drugs [38]–[42]. Different observations were made in these studies. In our study we reported that high-dose atorvastatin leads to a reduction in newly emerging CNS lesions and is associated with increased IL-10 secretion [39]. One recent trial demonstrated the neuroprotective properties of HMGCRI by showing a reduction in the rate of brain atrophy during a 3 year follow-up period [36]. Furthermore, some trials investigating HMGCRI in combination with interferon beta-1a did not report on any beneficial effect [41], [43], [44], one study with a small cohort of MS patients even reported on an increase in disease activity [38].

In our study, we provide evidence that iDC generated in the presence of atorvastatin (aiDC) support the expansion of suppressor T cells. Recently, Weber et al. reported that atorvastatin does not induce Th2 cells in vivo [45], in contrast to the observations made from ex vivo–restimulated myelin-specific T cells stemming from atorvastatin-treated EAE mice [12], [13]. In their study, Weber et al. also did not observe changes in the frequency of CD4+CD25+Foxp3+ Tregs in EAE animals following atorvastatin treatment but did observe an increase in IL-10 secretion [45] possibly originating from the APC compartment.

From our T cell priming experiments with aiDC, we conclude that atorvastatin promotes the differentation of iDC that do not optimally prime T cells, both *in vitro* and *in vivo*. Patient-derived DC modified to be tolerogenic could be considered as a future therapeutic strategy in autoimmune disease. Interestingly, two recent studies in experimental autoimmune myasthenia gravis [46] and experimental autoimmune neuritis [47] revealed the therapeutic benefit of intraperitoneally applying DC that were pre-treated with atorvastatin in culture. Both studies also report on the generation of CD4+ Foxp3+ Tregs and donwregulation of Th1/Th17 cytokines [46], [47].

The observed differential influence of aiDC on alloantigen-specific versus peptide-specific T cell responses could be explained by the mechanistic diversity in antigen processing utilized by the two different reactions; while alloantigen requires cytoskeleton-dependent endolysosomal processing [48], peptides do not require any intracellular processing prior to presentation. It is therefore our understanding, that OVA peptide loaded aiDC are in a position to bypass the influence of atorvastatin on antigen uptake and processing. In line with this and our observations regarding perturbations of the actin cytoskeleton in aiDC, we observed a reduced propensity of aiDC to reach their target organ (draining lymph nodes), which would partially explain the reduced capacity of OVA peptide loaded aiDC to prime T cells *in vivo*.

In this study we observed that human iDC were more sensitive to atorvastatin than murine iDC; lower doses of atorvastatin were sufficient to inhibit actin polymerization and expression of surface markers such as CD11c and CD11b in human iDC. We have previously observed the same phenomenon in antigen-specific T cells where atorvastatin appeared to influence human T cells at lower doses than murine T cells [12]. One factor that could explain this is the basal metabolic rate of both organisms, which is about seven times higher in mice than in humans [49].

Taken together, we found that the HMGCRI atorvastatin – that causes considerable alterations in cytoskeletal dynamics during iDC development – consequently alters the surface expression and localization of receptors and ligands that ultimately determine the decision between T cell priming or tolerance. While the relocalization of CD11c to the intracellular compartment requires further in depth investigation, we believe that by targeting CD40 expression, atorvastatin sets off a battery of downstream events that are characteristic of tolerogenic DC. Indeed we observed an increased production of IL-10, a decreased expression of costimulatory molecules on aiDC, a reduced ability of these cells to present antigen to T cells while still being able to migrate into T cell areas of secondary lymphoid organs – notwithstanding changes in the cytoskeleton – to induce T cell anergy and generate CD4+ T cell populations with a regulatory phenotype. Considering the pleiotropic nature of HMGCRI such as atorvastatin, we cannot exclude the involvement of several mechanisms that act together or independently to orchestrate the observed changes in functions (DC migration and T cell priming). For instance, we cannot completely attribute the functional changes observed (eg. T cell priming) solely to changes in surface expression of specific molecules. Indeed the cytoskeleton of DC and T cells determines the DC-T cell contact duration during T cell priming [50] and could be one target for atorvastatin that alters the T cell response. We believe that atorvastatin alters complex intracellular pathways – such as those related to the cytoskeleton [15] – to finally precipitate functional changes such as decreased migration or decreased priming capacity.

In summary, our study provides further evidence of atorvastatin's capacity to modulate the antigen-presenting cell compartment, resulting in morphological as well as functional changes in these cells that are connected with modulation of functional properties of T cells mediated by the APC-T cell interaction. This interaction plays a crucial role in different pathologies pertaining to allergy, autoimmunity or transplantation and could therefore be of therapeutic relevance in these clinical situations.

## Supporting Information

Figure S1 **Atorvastatin reduces** ***in vivo*** **migratory capacity of iDC, but does not influence their capacity to present peptide to antigen-specific T cells** ***in vitro*** **.** (A) Atorvastatin treated and untreated iDC generated from RFP-fluorescent mice were injected into C57BL/6 mice. The percentage of RFP-fluorescent iDC and aiDC that had migrated to the draining lymph nodes 24 h after injection was determined by FACS. Data from 5 mice are shown as mean percentage RFP+ cells (± SEM) of CD11c+CD11b+ cells within the harvested lymph nodes. (B) Naïve T cells from OTII-transgenic mice were labelled with CFSE and stimulated with OVA_323-339_ –loaded iDC or aiDC generated in the absence or presence of different concentrations of atrovastatin. CFSE-intensity was measured by flow cytometry after 72 h and percentage of divided T cells was analyzed. One representative experiment of three is shown.
(TIF)

Figure S2 **Dendritic cells generated in the presence of atorvastatin show an anti-inflammatory cytokinic profile.** Soluble cytokines from supernatants of DC generated in the absence or presence of atorvastatin were measured with FlowCytomix Multiplex (eBioscience). The expression of each cytokine is given as mean concentration of 2 experiments (pg/ml) ±SD.
(TIF)
