# Supplementary figures and images for: Modulation of Dendritic Cell Immunobiology via Inhibition of 3-Hydroxy-3-Methylglutaryl-CoA (HMG-CoA) Reductase
Source: PLoS One. 2014 Jul 11;9(7):e100871. doi: 10.1371/journal.pone.0100871 (PMC4094470; doi:10.1371/journal.pone.0100871)

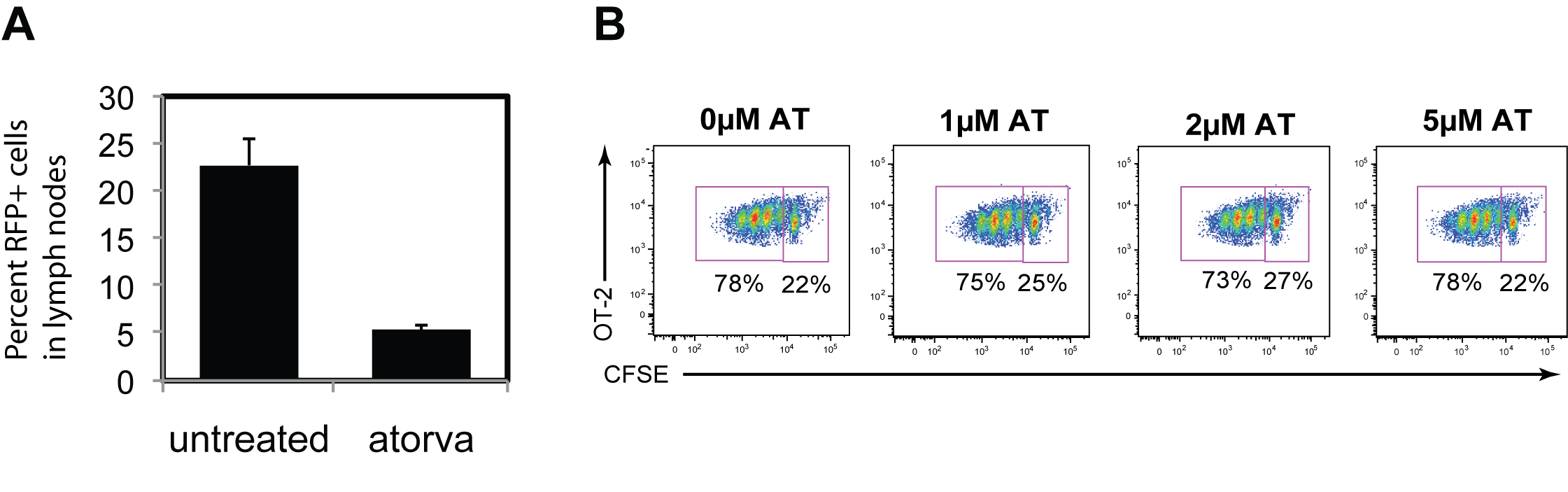

Supplement: Figure S1 — Atorvastatin reduces in vivo migratory capacity of iDC, but does not influence their capacity to present peptide to antigen-specific T cells in vitro . (A) Atorvastatin treated and untreated iDC generated from RFP-fluorescent mice were injected into C57BL/6 mice. The percentage of RFP-fluorescent iDC and aiDC that had migrated to the draining lymph nodes 24 h after injection was determined by FACS. Data from 5 mice are shown as mean percentage RFP+ cells (± SEM) of CD11c+CD11b+ cells within the harvested lymph nodes. (B) Naïve T cells from OTII-transgenic mice were labelled with CFSE and stimulated with OVA323-339 –loaded iDC or aiDC generated in the absence or presence of different concentrations of atrovastatin. CFSE-intensity was measured by flow cytometry after 72 h and percentage of divided T cells was analyzed. One representative experiment of three is shown. (TIF) [file pone.0100871.s001.tif]

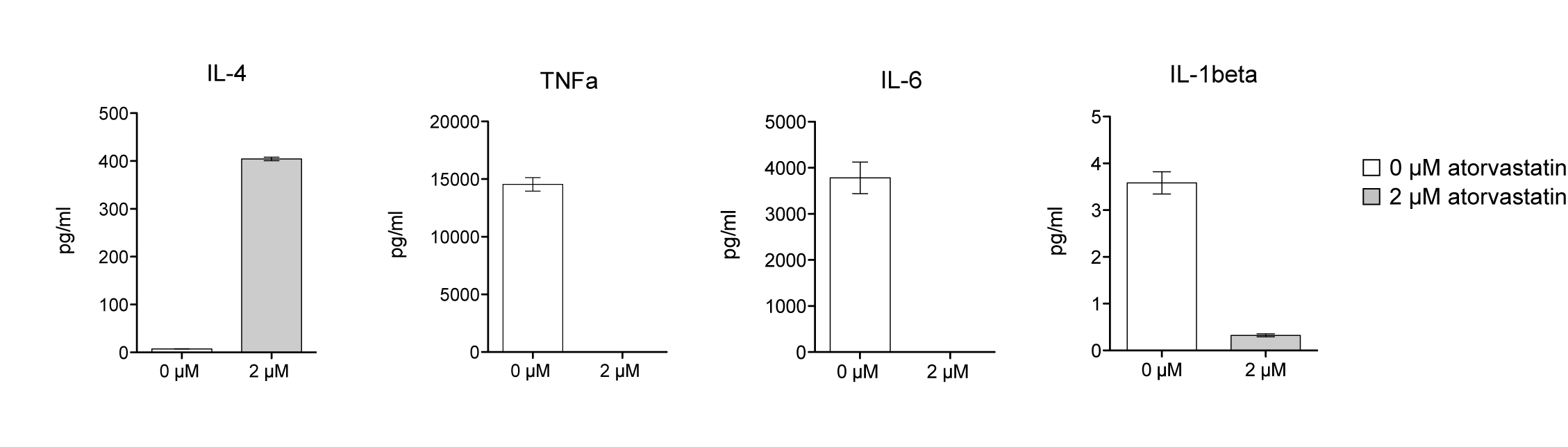

Supplement: Figure S2 — Dendritic cells generated in the presence of atorvastatin show an anti-inflammatory cytokinic profile. Soluble cytokines from supernatants of DC generated in the absence or presence of atorvastatin were measured with FlowCytomix Multiplex (eBioscience). The expression of each cytokine is given as mean concentration of 2 experiments (pg/ml) ±SD. (TIF) [file pone.0100871.s002.tif]
